# Polymorphisms in Manganese Transporters *SLC30A1*0 and *SLC39A8* Are Associated With Children's Neurodevelopment by Influencing Manganese Homeostasis

**DOI:** 10.3389/fgene.2018.00664

**Published:** 2018-12-20

**Authors:** Karin E. Wahlberg, Stefano Guazzetti, Daniela Pineda, Susanna C. Larsson, Chiara Fedrighi, Giuseppa Cagna, Silvia Zoni, Donatella Placidi, Robert O. Wright, Donald R. Smith, Roberto G. Lucchini, Karin Broberg

**Affiliations:** ^1^Division of Occupational and Environmental Medicine, Lund University, Lund, Sweden; ^2^Azienda USL-IRCCS di Reggio Emilia, Reggio Emilia, Italy; ^3^Institute of Environmental Medicine, Karolinska Institutet, Solna, Sweden; ^4^Radiological Sciences and Public Health, University of Brescia, Brescia, Italy; ^5^Icahn School of Medicine at Mount Sinai, New York, NY, United States; ^6^Microbiology and Environmental Toxicology, University of California, Santa Cruz, Santa Cruz, CA, United States

**Keywords:** manganese, neurotoxicity, neurodevelopment, ADHD, SLC30A10, SLC39A8

## Abstract

**Background:** Manganese (Mn) is an essential element but at excessive levels, it is neurotoxic. Even a moderate increase in Mn has been suggested to interfere with neurodevelopment in children. Genetics influencing Mn concentrations and toxicity is unclear.

**Objective:** We assessed, in a cross-sectional study, whether common single-nucleotide polymorphisms in the Mn transporters SLC39A8 (influx) and SLC30A10 (efflux) are associated with neurodevelopment in children.

**Design:** We genotyped *SLC39A8* (rs13107325 C/T) and *SLC30A10* (rs1776029 G/A and rs12064812 T/C) in Italian children (*n* = 686, ages 11–14). We then used linear regression models to analyze associations between genotype, blood Mn concentrations, and neurodevelopmental outcomes including intelligence, behavior, motor function, and sway. Inferred causal relationships were evaluated using instrumental variables (IV) analysis.

**Results:** For *SLC30A10* rs1776029, the minor allele (A) was associated with increased average blood Mn of 41% (*p* < 0.001), whereas minor alleles for rs12064812 (C) and rs13107325 (T) were associated with reduced blood Mn of 7% (*p* = 0.002) and 15% (*p* < 0.001), respectively. For children carrying genotypes associated with high blood Mn, we observed lower performance for certain IQ subtests, increased sway, and increased scores for behavioral problems. High Mn genotypes showed odds ratios of 2–4 *(p* ≤ 0.01) for high scores in tests assessing ADHD-related behavior. IV analyses suggested that several of the associations were mediated by blood Mn.

**Conclusions:** Our results suggest that common polymorphisms in *SLC39A8* and *SLC30A10* influence neurodevelopmental outcomes in children via differences in Mn homeostasis.

## Introduction

Diet is the major source of the essential element manganese (Mn) and since Mn is naturally abundant in several foods, e.g., whole grains, leafy vegetables, teas, and nuts, Mn deficiency is uncommon. However, excess intake of Mn can occur as a result of specialized diets, elevated levels in groundwater (from Mn leached from minerals), occupational exposure, and industrial pollution (Lucchini et al., 2014). Mn is involved in numerous enzymatic processes, such as protection against oxidative stress via Mn-dependent mitochondrial superoxide dismutases (Reddi et al., 2009) and the conversion of glutamate to glutamine in the brain (Takeda, 2003). Mn is therefore important for normal brain development, but it can also become neurotoxic at elevated levels (Horning et al., 2015). The range between essential and toxic doses of Mn is narrow and both ends of the spectrum have been associated with negative effects on children's neurodevelopment (Claus Henn et al., 2010). Several cross-sectional or small studies have demonstrated associations of environmental Mn exposure with cognitive, motor, and behavioral deficits (Bouchard et al., 2011; Menezes-Filho et al., 2011; Khan et al., 2012; Chiu et al., 2017), but a recent large prospective birth cohort study from Bangladesh did not find support for adverse effects of Mn exposure on neurodevelopment (Rahman et al., 2017). One reason for the conflicting results may be that intrinsic factors regulating Mn homeostasis influence how the body handles excess Mn and in turn Mn toxicity.

Elevated Mn concentrations can occur as a consequence of rare genetic variation affecting proteins involved in homeostatic regulation of Mn (reviewed in Chen et al., 2014). The cation influx transporter *SLC39A8* (ZIP8) has a high affinity for Mn but can also transport iron (Fe), zinc (Zn), and cadmium (Cd; He et al., 2006; Wang et al., 2012). Mutations in *SLC39A8* have been associated with severe Mn deficiency and related impairment of glycosylation (Park et al., 2015), as well as neurological and metabolic disorders (Speliotes et al., 2010; Teslovich et al., 2010; Carrera et al., 2012). SLC30A10 is a cell-surface protein with high expression in brain and was initially identified as a Zn transporter, but recently studies have demonstrated a specificity of SLC30A10 for Mn (Leyva-Illades et al., 2014; Zogzas et al., 2016). Rare loss-of-function *SLC30A10* mutations cause severely elevated Mn concentrations in the blood and brain, even in absence of external Mn exposure (Quadri et al., 2012; Tuschl et al., 2012; Lechpammer et al., 2014), resulting in neurodegenerative symptoms (Quadri et al., 2012; Tuschl et al., 2012).

Recently, we demonstrated that more common genetic variation in *SLC30A10* and *SLC39A8* also influence Mn concentrations in healthy individuals. Non-coding *SLC30A10* single-nucleotide polymorphisms (SNPs) rs12064812 and rs2275707 were associated with blood Mn concentrations in adults and neurological functions (sway and finger tapping speed) in elderly (Wahlberg et al., 2016). Further, we found that *SLC39A8* influences Mn concentrations in postnatal dentine (Wahlberg et al., 2017). The influence of SNPs in *SLC39A8* and *SLC30A10* on Mn regulation was also highlighted in a recent genome-wide association study, which identified two genetic variants, rs13107325 in *SLC39A8* and rs1776029 in *SLC30A10*, that were significantly associated with blood Mn concentrations (Ng et al., 2015). However, whether genetic variants in *SLC39A8* or *SLC30A10* also influence the regulation of Mn concentrations in children, or their neurodevelopment, remains unknown.

**Figure 1:**
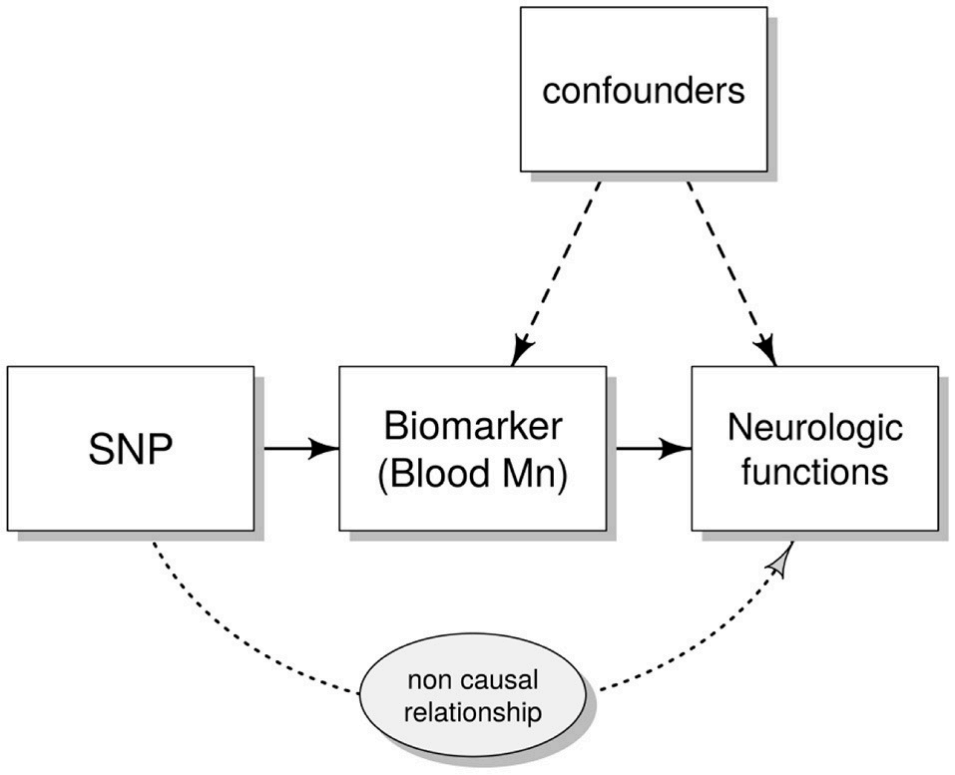
Directed acyclic graph (DAG) showing the instrumental variables (IV) assumptions underpinning a Mendelian randomization study. Single nucleotide polymorphism (SNP) represent the IV that (possibly) affects neurological functions only through the modifications induced in the biomarkers [the endogenous variable e.g., blood manganese (Mn)]. Confounders are variables that could influence both the biomarkers and the outcome of the neuro-developmental tests (e.g., age and gender).

Here we analyzed SNPs in *SLC39A8* (rs13107325) and *SLC30A10* (rs2275707, rs12064812 and rs1776029) previously associated with blood Mn in adults (Ng et al., 2015; Wahlberg et al., 2016). We examined these SNPs in a large cohort of children (*n* = 686) aged 11–14 years and studied the association of genotypes with blood Mn concentrations and children's performance in a comprehensive set of neurological tests including assessments of intelligence, behavior, balance and motor function. We used both ordinary linear regression and instrumental variables (IV) analyses (Figure 1); IV analyses allow the assessment of the causal effect of a risk factor on a disease outcome, using genetic determinants of the risk factor as IVs (Smith and Ebrahim, 2003; Pierce et al., 2013).

## Subjects and Methods

Detailed descriptions of methods for measurements of metals and neurological outcomes are presented in Supplementary Materials and Methods.

### Study Population

In this study we used a cross-sectional design. The complete study population consisted of 719 children, ages 11–14, residing in three different districts within the Province of Brescia, Italy: Valcamonica valley and Bagnolo Mella, which have experienced increased Mn exposure due to the presence of ferromanganese alloy plants, and Lake Garda, which has no history of Mn exposure. Valcamonica has a long history of ferromanganese alloy plants, which were active from 1902–2001. In Bagnolo Mella, one plant has produced ferromanganese since 1973 and was still active during data collection. Study sites and recruitment have previously been described (Lucchini et al., 2012a; Bauer et al., 2017; Chiu et al., 2017).

Recruitment was performed through the public school system. Forms of consent were given to 1232 subjects; out of 870 children for which consent was collected (participation rate = 70.6%), 719 were enrolled, 135 were rejected based on inclusion/exclusion criteria, and 16 were excluded for age/district over number. The inclusion criteria included: (i) born in the study area from resident families living in the study area since the 1970s; (ii) living in the study area since birth; (iii) between 11 and 14 years of age. Exclusion criteria were: (i) pre-existing neurological, hepatic, metabolic, endocrine, and psychiatric conditions affecting neurodevelopment (to evaluate genetics influencing normal neurodevelopment); (ii) taking medications with known neuropsychological side-effects; (iii) clinically diagnosed motor impairment of hand and fingers; (iv) pre-existing clinical diagnosis of neurodevelopmental disorders, e.g., ADHD and autism (v) visual deficits not adequately corrected. Thus, based on these exclusion criteria, the study group should overall represent a neurologically normal group. A first round of recruitment, including 311 children from Valcamonica and Lake Garda, started in 2007 and was completed in 2010; data collection in this round included personal sampling of air, water, and soil, indoor and outdoor dust from participants' homes and schools, and blood. A second round of recruitment took place from 2010 to 2014 and included 408 children from Valcamonica, Lake Garda, and Bagnolo Mella. From the complete cohort of 719 children, DNA samples for genetic analyses were successfully collected from 686 children, which constituted the study cohort. Of these children, 248 were from Lake Garda, 259 from Valcamonica and 212 from Bagnolo Mella. A participant flow chart is presented in Supplementary Figure S1.

The study design, information about study aims, and forms for informed consent were reviewed and approved by the ethics committees of the local Department of Health of Valcamonica and Brescia, and Lund University, Sweden. The procedures were in accordance with the Helsinki Declaration.

### Measurements of Mn in Soil

Mn in surface soil was measured *in situ* using a portable X-ray Fluorescence instrument (Thermo Scientific Niton, model XL3t) or estimated by GIS Kriging (37%) as previously described (Lucchini et al., 2012a).

### Biological Sampling and Measurements of Mn, Lead, Fe, and Ferritin

Sampling and preparation of blood for analyses of Mn concentrations were conducted as previously described (Bauer et al., 2017).

Mn, lead (Pb) and Fe levels in blood were measured by magnetic sector inductively coupled plasma mass spectrometry or by Zeeman graphite furnace atomic absorption spectrometry, as described elsewhere (Apostoli et al., 2000; Smith et al., 2007; Lucas et al., 2015).

Ferritin was measured in plasma by chemiluminescent microparticle immunoassays using the Architect SR 2000 Immunoassay Analyzer (Abbott Diagnostics, Illinois, USA).

### Assessment of Cognitive Behavioral Functions

#### Intelligence

Children's IQ was assessed using the Wechsler Intelligence Scale for Children (WISC), third edition which includes a panel of subtests (Supplementary Table S2) and gives an estimation of the child's total IQ. Raw scores of each scale are corrected for age in weighted scores from 1 (worse score) to 19 (best score). Total IQ is calculated based on scores for 10 subtests.

#### Motor Function

Children's motor function was assessed by a panel of tests covering different aspects of motor function, as previously described in detail (Lucchini et al., 2012a; Chiu et al., 2017) and included the 5 subtests of the Luria-Nebraska Motor Battery, finger tapping speed assessed by the Swedish Performance Evaluation System (SPES; Iregren et al., 1996) and reaction time evaluated by the SPES version of Visual Simple Reaction Time Test.

#### Sway/Tremor

Children's tremor was assessed with Tremor 7.0 of Danish Products developments (Després et al., 2000) and body sway (sway area, intensity, velocity, and mean sway) was measured using a balance plate.

Hand dexterity and perceptual speed was assessed using the Pursuit Aiming test (Fleishman, 1954).

#### Behavior

Children's behavior was assessed using the Conners' Adolescent Self-Report Scale (CASS), which includes 10 subscales: family problems, emotional problems, conduct problems, cognitive problems/inattention, anger control problems, hyperactivity, attention deficit hyperactivity disorder (ADHD) index, Diagnostic and Statistical Manual of Mental Disorders, 4th Edition (DSM-IV) of inattention, DSM-IV of hyperactivity/impulsivity, and DSM-IV Total (total score of the 2 subscales assessing inattention and hyperactivity/impulsivity; Lucchini et al., 2012b). Of the DSM-IV scales, only DSM-IV total was included in this study for assessment of ADHD-related behavior (Wolraich et al., 2011).

Approximately half of the children were also assessed by revised short versions of the Conners' Parent's Rating Scales (CPRS-R) and Teachers Rating Scales (CTRS-R) which included four subscales (oppositional problems, cognitive problems/inattention, hyperactivity, and ADHD-index; Conners et al., 1998a,b).

Raw scores were corrected for age and sex, and converted into T-scores, which are standardized scores with a mean of 50 and a standard deviation of 10. As a rule, T-scores above 56 indicate a borderline problematic picture and scores over 60 are cause for concern. The validated Italian versions of Conner's Rating Scale-Revised (CPRS-R, CTRS-R, CASS) were used for the assessment (Conners, 2001).

### Genotyping

DNA was extracted from whole blood samples using the QIAamp DNA Blood Mini kit (Qiagen, Hilden, Germany). Genotyping was performed by TaqMan real-time PCR for rs2275707, rs12064812, and rs13107325 (Thermo Scientific assay IDs C_15879436_20, C_32155052_10, and C___1827682_10, respectively), as previously described (Wahlberg et al., 2016, 2017). Reactions were analyzed on the ABI 7900HT Fast Real Time PCR System (Applied Biosystems, Thermo Fisher, Waltham, USA), using the manufacturer's recommended standard conditions.

Rs1776029 was genotyped by pyrosequencing as described in Wahlberg et al. (2017). The PCR product was purified using Streptavidin Sepharose High Performance beads (Amersham Biosciences, UK) and pyrosequencing was carried out using the PyroMark reagents and PSQ HS96 Pyrosequencing System (Qiagen) according to the manufacturer's protocol. For quality control of genotyping data, >5% of samples were re-analyzed for all SNPs in a separate round of experiments with a 100% agreement between duplicates. Data quality was also assessed by evaluating Hardy-Weinberg equilibrium using the conventional Chi-Square test.

### Statistical Analyses

We tested, by linear regression models and IV analysis, our hypothesis that polymorphisms in Mn transporters are associated with blood Mn concentrations and that carriers of genotypes associated with higher Mn in blood perform worse in tests for neurodevelopment.

Due to the strong linkage between the SNPs rs2275707 and rs1776029 in *SLC30A10* and the fact that out of the two, rs1776029 showed stronger associations with blood Mn (Results section), we included only rs1776029 in analyses of associations with neurological parameters. Moreover, due to the low representation of the rs13107325 minor homozygotes (TT), these individuals were combined with heterozygotes in the statistical analyses.

Linear regression models were used to evaluate associations between genotypes and Mn concentrations in blood. Blood Mn was log-transformed to fit the model assumptions. The models were adjusted for possible influential factors, including age, gender, ferritin (stored iron) concentrations, and environmental Mn exposure by soil Mn concentrations (log-transformed). Iron status can influence Mn concentrations, probably due to competition between Fe and Mn uptake (Kippler et al., 2009). Higher soil Mn was found in regions of Valcamonica and Bagnolo Mella compared to Lake Garda (Borgese et al., 2013; Pavilonis et al., 2015), indicating that soil Mn is a suitable marker of cumulative/historical environmental exposure. Partial eta squared was obtained from the models for estimation of effect size of the genotype variables on blood Mn.

Associations between genotypes and IQ, motor function, sway, tremor, and behavior were studied using multiple linear regression analysis (ordinary least squares; OLS) taking into account the effect of possible confounders. Sway, tremor, and Conners' scores were log-transformed to better fit the linear model assumptions. OLS models for neurological parameters were, in addition to gender, age, ferritin, and Mn in soil (log-transformed), also adjusted for blood Pb concentrations, body mass index (BMI), drinking habits, socioeconomic status (SES), maternal education, and parity. Pb is a known neurodevelopmental toxicant (Lucchini et al., 2012b). BMI, SES, parity, and maternal education are well documented confounders for performance during neurological testing in children (Eriksen et al., 2013; Goldberg et al., 2014; Tabriz et al., 2015; von Stumm and Plomin, 2015). Cohort characteristics of these variables are presented in Supplementary Table S2. We also adjusted for test-examiners for all neurological outcomes except behavior due to the lack of involvement of test-examiners in the Conners' scales.

Logistic regression was used to estimate odds ratios for high (≥56) vs. low (< 56) scoring on the Conner's Adolescents Self-Report Scale subscales for ADHD-index and DSM-IV total according to genotypes.

We tested by Mendelian randomization (MR) analysis our hypothesis that Mn in blood is the causal factor for the associations between Mn transporter polymorphisms and neurodevelopment. MR is used to infer the causal relationships between an exposure and an outcome and makes use of instrumental variables (IV), represented here by a genetic score based on the SNPs involved in Mn homeostasis. The genotype score was developed based on genotypes of all SNPs, where rs1776029 was encoded as 0, 1, or 2 for GG, GA, and AA, respectively; rs1776029 was encoded as 0 for CC/TC and 1 for TT; and rs13107325 was encoded as 0, 1, or 2 for TT, CT, and CC, respectively. The scores were added up to obtain a total genotype score, ranging from 0 to 5.

For an IV to be valid, the following conditions are assumed: the genetic variant (here genotype score) is causally associated with the exposure (Mn in blood) and affects the outcome only through the exposure and not through any other causal pathway. Furthermore, the IV should not be associated with any confounders of the association between the exposure and the outcome (Figure 1; Burgess et al., 2017).

The IV analysis was performed using a two-stage least squares approach as implemented in R using the AER package (Kleiber and Zeileis, 2008). If the IVs are valid, the IV estimate represents the causal component of the observed association. The genotype scores were selected as IVs because they showed strong associations with blood Mn. The Durbin-Wu-Hausman test was used to detect endogeneity and to decide when to reject the hypothesis of consistency of the OLS estimates and thus prefer the IV over the OLS model. A “weak instruments” test was also performed to ensure the validity of the IV estimates.

We did not correct for multiple comparisons in theses analyses, but rather focused on patterns of associations between genotype, Mn, and neurodevelopmental outcomes. Statistical analyses and graphics were performed using the program R v.3.4.1 (R Core Team, 2017).

## Results

### Study Population Characteristics and Exposure Measurements

The characteristics of the study cohort (individuals with DNA samples) are presented in Table 1. The age groups represented were 10 (0.6%), 11 (22.2%), 12 (32.7%), 13 (36.2%), and 14 (8.5%) years of age. There were 202 children (29%) with BMI above 22 and 55 (8%) with BMI above 27 that could be considered overweight and obese, respectively. Minimum and maximum values for blood Mn concentrations were 4.0 and 34.3 μg/L, thereby representing 8.4-fold variation. Based on previous studies, blood Mn concentrations were in the normal range (Lucchini et al., 2014) while blood Pb levels were in the lower range (Lucchini et al., 2012b). Mn in soil varied between 124 ppm (min) and 17,000 ppm (max), thereby representing 137-fold variation. There was no correlation between Mn in soil and children's blood Mn concentrations (correlation coefficient Spearman's rho = −0.015, *p* = 0.690).

**Table 1:** Study cohort characteristics.

**Variable**	***n***	**Median**		**Percentile (5^**th**^, 95^**th**^)**
***n*** **=** **686 CHILDREN (360 BOYS AND 326 GIRLS)**				
Age	686	12		11, 14
BMI	643	19.9		15.6, 29.2
Blood Mn (μg/L)	681	10.9		6.5, 17.0
Blood Pb (μg/L)	681	13.3		7.2, 32.0
Ferritin (ng/mL)	683	27.0		8.0, 70.0
Mn in soil (ppm)	666	706		267, 1898
MAF rs1776029 (G/A)	676		22%	
MAF rs2275707 (A/C)	685		22%	
MAF rs12064812 (T/C)	685		31%	
MAF rs13107325 (C/T)	682		9%	

*MAF, minor allele frequency; ppm, parts per million*.

An overview of children's performance in neurological testing is presented in Supplementary Tables S2–4 and social/life style covariates included in statistical models in Supplementary Table S5. Overall, the neuro-functional outcomes show normal performance levels, although a small percentage of children exhibit borderline intelligence scores.

### Characteristics of the Genetic Variants

*SLC30A10* rs1776029 (G/A) is situated approximately 7.6 kb downstream of the *SLC30A10* transcript and *in silico* analysis of the genomic sequence surrounding the SNP using publicly available data (USCS Genome Browser; https://genome.ucsc.edu/, accessed April 2017) revealed that this region displays noticeable signs of regulatory activity, as indicated by strong histone acetylation, DNase I hypersensitivity, and transcription factor binding (Supplementary Figure S2A). Linkage analyses of the three *SLC30A10* SNPs in this population showed strong linkage between rs2275707 and rs1776029 (*R*^2^ = 0.85) but no linkage between rs12064812 and the other two SNPs (Supplementary Figure S2B).

All SNPs were in Hardy-Weinberg equilibrium. The minor allele frequencies (MAFs, Table 1) were in agreement with previous published genotyping data from the same area (Carrera et al., 2012; Wahlberg et al., 2016) and publicly available genotyping data at the NCBI SNP database (http://www.ncbi.nlm.nih.gov/snp, accessed April 2017).

### Mn Transporter Genotypes Are Associated With Blood Mn Concentrations

**Figure 2:**
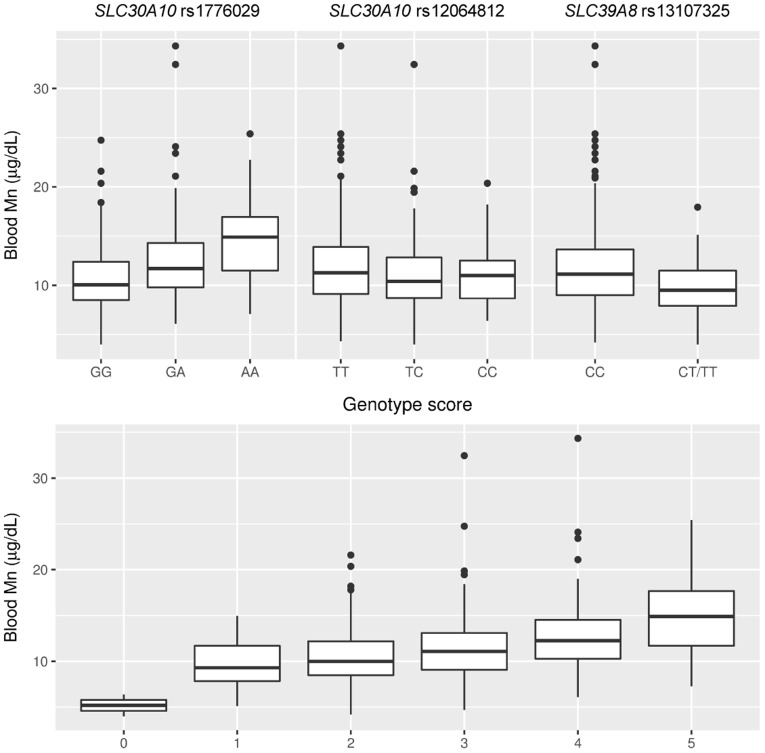
Associations of *SLC30A10* and *SLC39A8* genotypes and a combined genetic score of these genotypes with blood Mn concentrations.

Genotypes for each of the four SNPs were analyzed in association with Mn concentrations in blood (Figure 2; Supplementary Table S1). We observed strong positive associations with blood Mn for the *SLC30A10* rs1776029 minor allele A (41% higher Mn AA vs. GG, *p* < 0.001, β AA vs. GG = 0.14, CI 0.10, 0.18) and rs2275707 rare allele C (29% lower Mn CC vs. GG, *p* < 0.001, β CC vs. AA = 0.10, CI 0.06, 0.15). In contrast, the *SLC30A10* rs12064812 minor allele (C) was associated with decreased blood Mn concentrations (7%, *p* = 0.008, β CT vs. TT = −0.03, CI −0.05, −0.01).

The *SLC39A8* rs13107325 minor allele homozygotes (TT, *n* = 3) showed 52% lower average blood Mn concentrations compared to the major allele homozygotes (CC). One of the TT carriers had the lowest blood Mn of the entire cohort (4.0 μg/L). Carriers of the T allele showed an average of 15% lower blood Mn concentrations compared to major allele homozygotes (*p* < 0.001, β CT/TT vs. CC = −0.07, CI −0.10, −0.05; Figure 2, Supplementary Table S1). The genotype score showed a clear dose-response in Mn concentrations in blood (Figure 2). There was no association of rs13107325 with Fe concentrations in blood (Kruskal Wallis test, *p* = 0.894).

Estimates of effect size showed a contribution of 10% (Eta-squared) for rs1776029, 1.5% for rs12064812, and 6.4% for rs13107325 on total variance in blood Mn concentrations in the cohort and the three genetic variants together accounted for 17.9% of the total variance. The genotype score contributed to 13% of the variance in blood Mn. Other than genotype, which had the strongest influence on blood Mn in the statistical models, ferritin was also significantly associated with blood Mn but gender, age, or Mn in soil showed no association. The two SNPs in *SLC30A10* were not significantly associated with potential confounding factors such as blood Pb concentrations, BMI, or age, supporting the use of these SNPs in a genotype score as instrumental variables for IV analysis. The Mn-raising allele of rs13107325 in *SLC39A8* was associated with lower BMI (*p* = 0.002, CC vs. CT/TT) but was not significantly associated with other potential confounders.

### Mn Transporter Genotypes and Outcomes of Neurological Testing

Mn transporter genotypes were analyzed in association with outcomes of a comprehensive set of neurological tests aiming to assess IQ, motor function, sway, tremor, and behavior (Supplementary Tables S6, 7).

#### IQ

**Figure 3:**
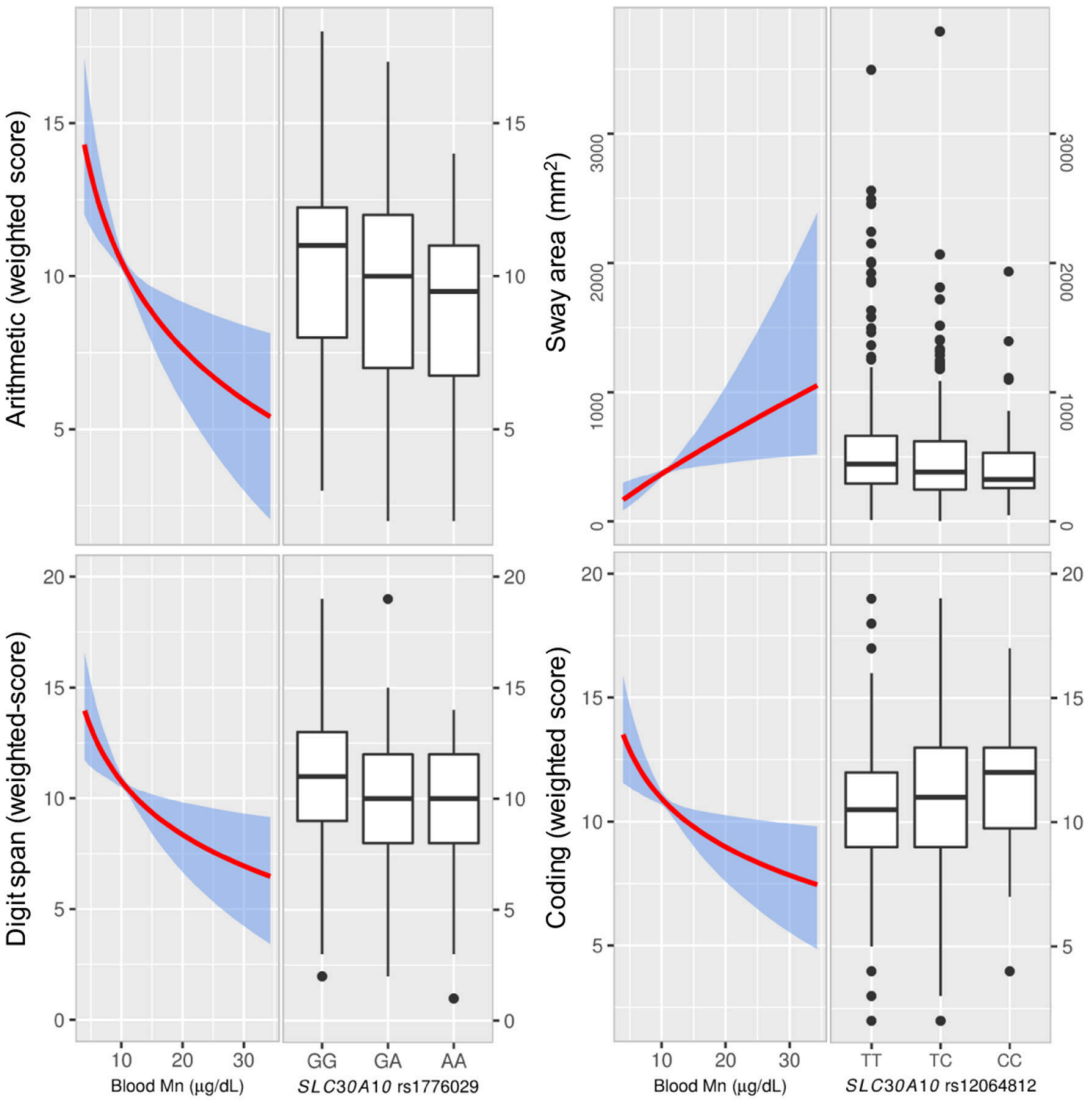
The effect of blood Mn estimated from the instrumental variable (IV) analyses and associations of *SLC30A10* genotypes with neurological testing. Left graphs show the effect of blood Mn estimated from the IV analyses with the genetic score as the IV (with 95% bootstrapped confidence band for the regression estimates) on neurological testing for IQ (Wechsler scales) and sway. The right graphs show the distribution of neurological testing for the various allelic combinations of the SNPs that showed the strongest association with the outcome. The *SLC30A10* rare allele A, which was associated with higher blood manganese (Mn) showed reduced scoring for Wechsler subscales arithmetic and digit span. IV-analysis showed significant negative associations between blood Mn and scoring. We also observed associations for *SLC30A10* rs12064812 genotypes with coding and sway (sway area), where the rare allele C, which was associated with lower blood Mn, was associated with higher coding and reduced sway. The IV analyses of blood Mn vs. coding and sway were significant. Manganese, Mn; instrumental variable, IV.

For total IQ, we observed no evident associations with genotypes. However, there was a general trend for several subscales with genotypes related to high blood Mn being associated with reduced performance and vice versa; the rs1776029 minor allele (A; higher blood Mn) was associated with reduced performance for arithmetic (β AA vs. GG = −1.21, CI −2.41, −0.01; *p* = 0.006) and digit span (β AA vs. GG = −0.90 CI −1.97, 0.18: *p* = 0.011; Figure 3). In contrast, the *SLC30A10* rs12064812 minor allele (C; lower blood Mn) showed associations with improved performance for digit span (β CC vs. TT = 1.06 CI 0.23, 1.89; *p* = 0.053) and coding (β CC vs. TT = 0.96 CI 0.17, 1.76; *p* = 0.053) For *SLC39A8* rs13107325 we observed no association between genotypes and IQ (Supplementary Table S6).

#### Motor Function and Sway/Tremor

Motor function assessed by the sum of the 5 Luria subscales (Luria sum) was associated with rs1776029 (β AA vs. GG = −6.10 CI −11.48, −0.99; *p* = 0.004). We also observed associations of genotypes with balance; the rs12064812 minor allele (C; lower blood Mn) was negatively associated with sway area (β CC vs. TT = −0.09 CI −0.20, 0.01; *p* = 0.010; Figure 3) and sway velocity (β CC vs. TT = −0.03 CI −0.09, 0.03; *p* = 0.048). There was also an association of the rs12064812 C allele with increased finger tapping (Supplementary Table S6), which is in agreement with previous findings in elderly subjects (Wahlberg et al., 2016).

No significant associations were observed for genotypes with tremor, pursuit aiming and reaction time (Supplementary Table S6).

#### Behavior

**Figure 4:**
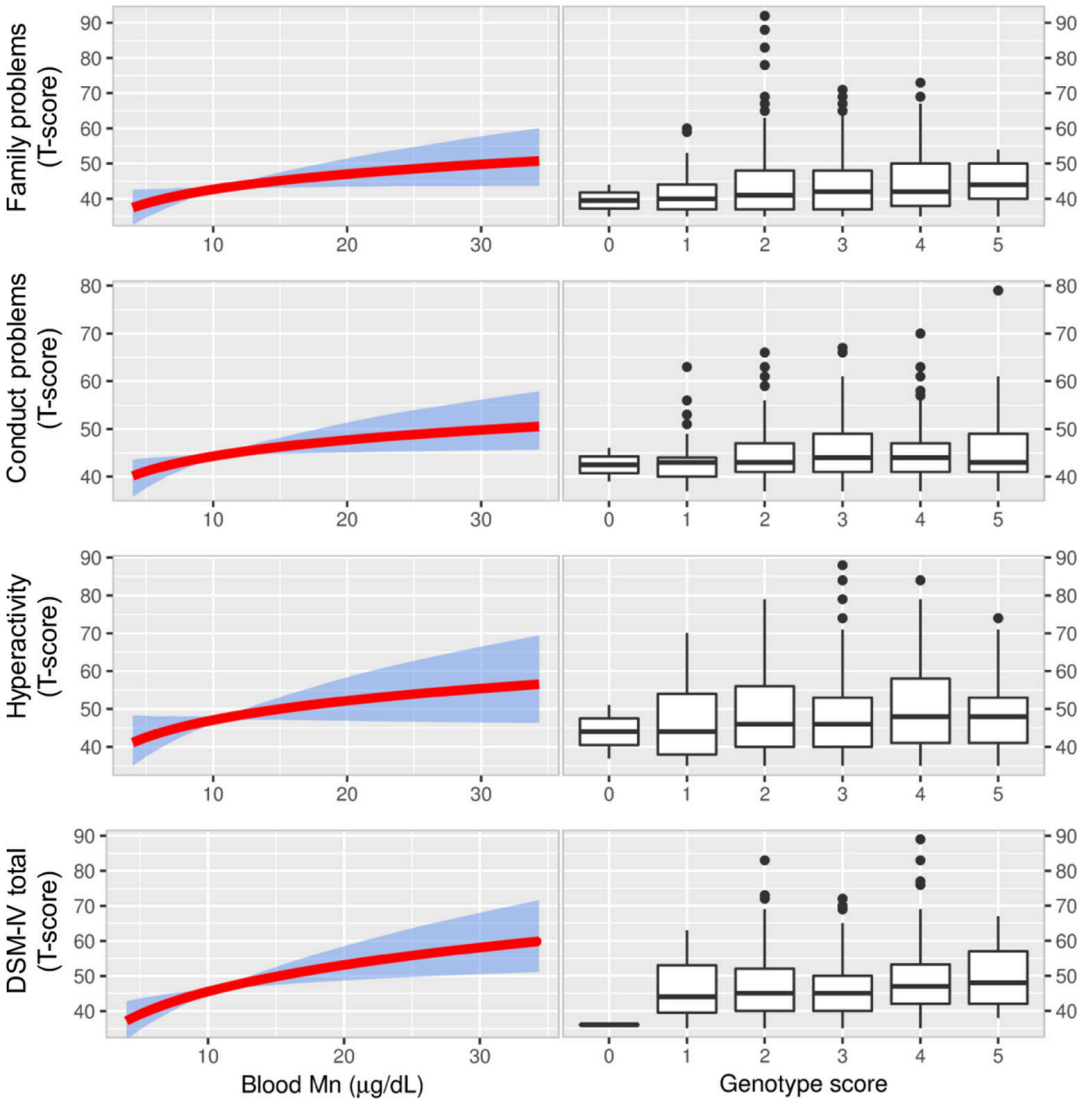
The figure displays associations between blood Mn and CASS outcomes from IV analyses (with 95% bootstrapped confidence band for the regression estimates) using the genetic score as the IV (left graphs). There was a pattern of positive associations between blood Mn and scoring for behavioral problems in IV-analyses. The most apparent associations were observed for DSM-IV total which is a combination of two tests for inattention and hyperactivity/impulsivity and used for ADHD diagnosis. As a reference we also show that scoring for the same behavioral problems were higher for genetic scores associated with higher blood Mn (right graphs). Manganese, Mn; Conners' Adolescent Self-Report Scale (CASS), Instrumental variable, IV; Diagnostic and Statistical Manual of Mental Disorders 4th Edition total score of inattention and hyperactivity/impulsivity, DSM-IV total.

Associations of genotypes with the Conners' Adolescents Self-Report Scale (CASS) showed a general trend of increased scoring (i.e., more problematic behavior) in association with genotypes that were also associated with increased Mn concentrations in blood and vice versa (Figure 4, Supplementary Table S7). The Parent's Rating Scales (CPRS-R) and Teachers Rating Scales (CTRS-R), for which only half of the children were tested, showed similar patterns to CASS but the associations were weaker (Supplementary Table S7).

We observed associations of the CASS subscales family problems, conduct problems, hyperactivity, and DSM-IV total with genotype (Figure 4, Supplementary Table S7). The rs1776029 minor allele (A; higher blood Mn) was associated with increased scoring for DSM-IV (β AA vs. GG = 0.03 CI 0.0003, 0.06; *p* = 0.019); in contrast, the rs13107325 minor allele (T; lower blood Mn) was associated with reduced scoring (β CT/TT vs. CC = −0.02 CI (−0.04, −0.004; *p* = 0.006). The rs1310725 T allele was also inversely associated with family problems (β CT/TT vs. CC = −0.02 CI −0.04, −0.004; *p* = 0.006) and hyperactivity (β CT/TT vs. CC = −0.02 CI (−0.04, 0.003; *p* = 0.039). The rs12064812 minor allele (C; lower blood Mn) was significantly inversely associated with conduct problems (β CC vs. TT = −0.01 CI −0.03, 0.003; *p* = 0.029).

The three children who were homozygotes for the rs13107325 minor allele (TT) showed a very distinct profile in CASS by scoring on average 10–20% lower compared with those who were homozygotes for the major allele (CC). Moreover, they were near or within the 5th percentile (atypical range) for several subtests. The group comprised one girl aged 12 and two boys aged 11 and 13. These three subjects did not show any deviant characteristics with respect to ferritin levels, environmental Mn exposure, BMI, SES, maternal education, alcohol consumption, or test examiner.

Generally, other than genotype, factors significantly contributing to the models for subtests related to IQ and behavior were sex, SES, and blood Pb. We also observed significant contribution of Mn in soil on some models with behavioral outcomes. For motor function and balance; sex, BMI and age (finger tapping only) were the main contributing factors on the models.

To further assess the significance of *SLC30A10* and *SLC39A8* genotypes for ADHD-related behavioral problems, we analyzed differences in risk between high- and low-scoring individuals for the CASS ADHD-index and DMS-IV total subscales by logistic regression. The cut-off between groups was set at 56, which represents the limit between normal and borderline problematic phenotype. We observed that genotypes associated with high blood Mn were also associated with increased risk for high scores in tests assessing ADHD-related behavior (i.e., Conners' subscales ADHD-index and DSM-IV total; Table 2).

**Table 2:** Adjusted odds ratios for high (≥56) vs. low (< 56) scoring on the Conner's Adolescents Self-Report Scale subscales for ADHD-index and DSM-IV total according to genetic variants associated with blood manganese levels.

	**ADHD-index**			**DSM-IV total**		
**Genotype**	***N*^a^**	**OR (95% CI)^b^**	***p***	***N*^a^**	**OR (95% CI)^b^**	***p***
***SLC30A10*** **rs1776029**
GG	39/319	1.00 (reference^c^)	–	46/312	1.00 (reference^c^)	–
GA	23/173	1.06 (0.60-1.86)	0.84	38/158	1.62 (1.00-2.62)	0.05
AA	5/22	1.43 (0.49-4.19)	0.51	9/18	3.08 (1.25-7.57)	0.01
***SLC30A10*** **rs12064812**
CC/TC	24/266	1.00 (reference^c^)	–	39/251	1.00 (reference^c^)	–
TT	43/248	2.10 (1.21-3.66)	0.008	54/237	1.51 (0.95-2.40)	0.08
***SLC39A8*** **rs13107325**
TT/CT	4/99	1.00 (reference^c^)	–	12/91	1.00 (reference^c^)	–
CC	63/415	4.16 (1.45-11.9)	0.008	81/397	1.68 (0.87-3.27)	0.12

*DSM-IV total, Diagnostic and Statistical Manual of Mental Disorders, 4th Edition total score of inattention and hyperactivity/impulsivity; OR, odds ratio; CI, confidence interval*.

a *Number of children with high (≥56) vs. low (< 56) scoring for ADHD-index and DSM-IV total*.

b *Adjusted for age, gender, body mass index, ferritin, and Pb levels, Mn in soil, drinking habits, Socioeconomic status, maternal education, and parity*.

c *Reference genotypes are associated with lowest manganese concentrations in blood*.

### Instrumental Variables Analysis of Mn Genotype and Neurological Outcomes

In addition to linear regression (OLS), we took advantage of the genetic score as an IV to assess blood Mn as the causal factor of observed associations between genotype and neurological outcomes.

For tests of neurological function including IQ, motor function, and balance, the IV analysis demonstrated a significant inverse association of blood Mn with coding (−6.35 CI −11.73, −0.97; *p* = 0.021), arithmetic (−7.92 CI −14.16, −1.68; *p* = 0.013) and digit span (−6.57 CI −12.14 −1.01; *p* = 0.021), and positive associations with sway area (0.75 CI 0.050, 1.46; *p* = 0.036) and sway velocity (0.33, CI −0.057, 0.71; *p* = 0.096; Supplementary Table S8, Figure 3).

Further, the IV analyses, but not OLS, generally showed a positive, and in several cases significant, influence of blood Mn on behavioral problems (Table 3, Figure 4). This was particularly evident for the CASS tests, but a similar pattern was found for the CPRS and CTRS tests.

**Table 3:** Instrumental variables analysis of genotype score and blood Mn with behavioral outcomes assessed by CASS, CPRS, and CTRS.

**Main scale**	**Subscale**	**Statistic**	**OLS**	**IV**
			**Blood Mn**	**Blood Mn**
CASS	Family problems	β (95% CI)^a^	0.019 (−0.031, 0.069)	0.22^***^ (0.052, 0.38)
		P^b^	*p =* 0.46	*p =* 0.01
	Emotional problems	β (95% CI)^a^	0.005 (−0.047, 0.058)	0.17^**^ (−0.002, 0.34)
		P^b^	*p =* 0.85	*p =* 0.053
	Conduct problems	β (95% CI)^a^	−0.009 (−0.042, 0.024)	0.16^***^ (0.025, 0.246)
		P^b^	*p =* 0.59	*p =* 0.016
	Cognitive problems/inattention	β (95% CI)^a^	−0.003 (−0.053, 0.047)	0.15^**^ (−0.009, 0.32)
		P^b^	*p =* 0.91	*p =* 0.065
	Anger control problems	β (95% CI)^a^	0.012 (−0.034, 0.058)	0.18^**^ (0.032, 0.33)
		P^b^	*p =* 0.62	*p =* 0.018
	Hyperactivity	β (95% CI)^a^	0.030 (−0.029, 0.089)	0.21^**^ (0.018, 0.40)
		P^b^	*p =* 0.32	*p =* 0.032
	ADHD-index	β (95% CI)^a^	0.012 (−0.038, 0.063)	0.24^***^ (0.073, 0.41)
		P^b^	*p =* 0.63	*p =* 0.005
	DSM-IV total	β (95% CI)^a^	0.035 (−0.014, 0.084)	0.29^***^ (0.12, 0.45)
		P^b^	*p =* 0.16	*p =* 0.001
CPRS	Oppositional	β (95% CI)^a^	0.027 (−0.045, 0.10)	0.12 (−0.077, 0.31)
		P^b^	*p =* 0.46	*p =* 0.24
	Cognitive problems/inattention	β (95% CI)^a^	−0.013 (−0.078, 0.053)	0.18^**^ (−0.008, 0.36)
		P^b^	*p =* 0.71	*p =* 0.062
	Hyperactivity	β (95% CI)^a^	0.008 (−0.045, 0.061)	0.13* (−0.017, 0.27)
		P^b^	*p =* 0.76	*p =* 0.085
	ADHD-index	β (95% CI)^a^	−0.017 (−0.092, 0.058)	0.13 (−0.073, 0.34)
		P^b^	*p =* 0.66	*p =* 0.21
CTRS	Oppositional	β (95% CI)^a^	−0.025 (−0.080, 0.030)	0.14** (−0.009, 0.28)
		P^b^	*p =* 0.38	*p =* 0.068
	Cognitive problems/inattention	β (95% CI)^a^	−0.029 (−0.097, 0.040)	0.12* (−0.061, 0.29)
		P^b^	*p =* 0.42	*p =* 0.20
	Hyperactivity	β (95% CI)^a^	−0.027 (−0.086, 0.033)	0.17*** (0.005, 0.329)
		P^b^	*p =* 0.38	*p =* 0.044
	ADHD-index	β (95% CI)^a^	−0.045 (−0.11, 0.024)	0.14** (−0.045, 0.32)
		P^b^	*p =* 0.21	*p =* 0.14

CASS, Conners' Adolescent Self-Report Scale; CPRS, Conners' Parent's Rating Scales; CTRS Conners' Teachers's Rating Scales; OLS, ordinary least squares; IV, instrumental variable; CI, confidence interval; ADHD, attention deficit hyperactivity disorder, ADHD; DSM-IV total, Diagnostic and Statistical Manual of Mental Disorders, 4th Edition total score of inattention and hyperactivity/impulsivity.

a *Adjusted for age, gender, body mass index, ferritin and Pb levels, Mn in soil, drinking habits, SES, maternal education, and parity*.

b p-values of associations between genotypes and outcome refers to overall p-value of genotype score variable. Asterisks(

* p < 0.10;

** p < 0.05;

*** *p < 0.01) after p-values for IV refer to p-value of Wu-Hausman test where a low p-value indicates that the IV model is more consistent than OLS, and thus, the more suitable model of the two*.

## Discussion

**Figure 5:**
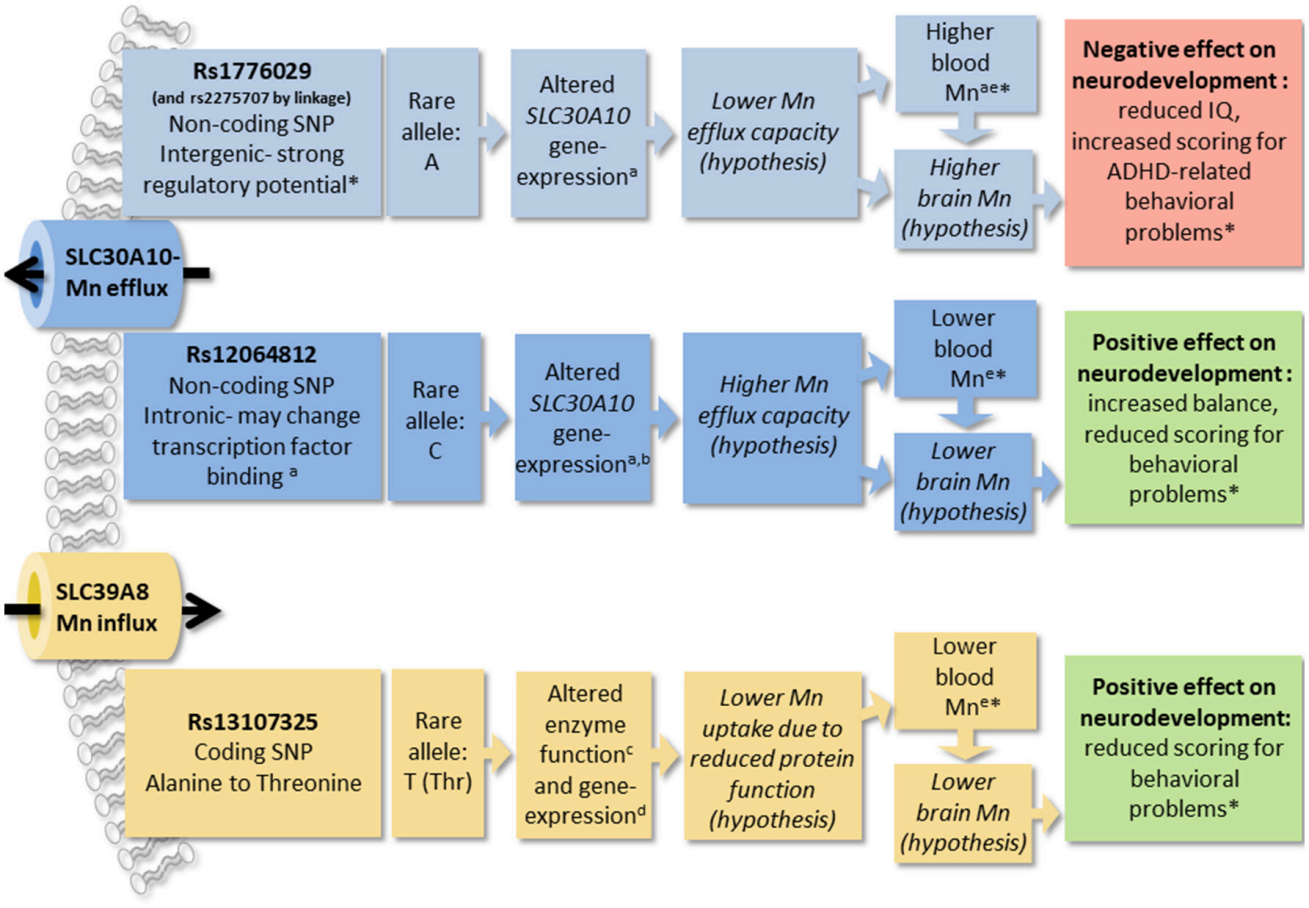
The figure summarizes results from this and other studies of SNPs in manganese (Mn) transporters SLC30A10 and SLC39A8 and prosed mechanisms of effects. Findings from this study are marked with asterisks (*). ^a^(Wahlberg et al., 2016); ^b^https://gtexportal.org/home/; ^c^(Zhang et al., 2016); ^d^(Speliotes et al., 2010); ^e^(Ng et al., 2015). Manganese, Mn; single nucleotide polymorphism, SNP; attention deficit hyperactivity disorder, ADHD.

In this study of healthy children, we show that common genetic variation in the Mn transporter genes *SLC39A8* and *SLC30A10* are associated with children's performance in neurological testing. We also show that the same SNPs are strong modifiers of blood Mn concentrations, together accounting for 17.9% of the total variation in blood Mn levels in the study cohort. Further, when using these SNPs as a genetic score in IV analysis, we find that several of the associations between the genetic score and neurological outcomes are mediated by blood Mn concentrations, supporting the hypothesis that variations in Mn levels during childhood may influence neurodevelopment. We propose that altered Mn homeostasis, including the regulation of Mn in the brain, is the underlying cause of the influence of Mn transporter SNPs on children's neurological performance in this study. A summary of results from this study and prosed mechanisms discussed below are presented in Figure 5.

SNPs in *SLC39A8* and *SLC30A10* have previously been associated with neurological outcomes; *SLC39A8* SNPs have been linked to schizophrenia (Carrera et al., 2012), chronic stress (Bruenig et al., 2014), and mental disability (Boycott et al., 2015), and *SLC30A10* genotypes were associated with neurological performance in elderly people (Wahlberg et al., 2016). However, this is to our knowledge the first study linking common SNPs in these transporters to children's neurodevelopment. The direction of the associations of SNPs in the two Mn transporter gene regions with blood Mn concentrations and neurological outcomes in this study are consistent with other studies in adults of the same SNPs (Ng et al., 2015; Wahlberg et al., 2016). Still, the results need to be interpreted cautiously, since adjustment for multiple comparisons was not performed and thus there is a possibility that some associations are false positives. Nevertheless, the interpretation of the results is based on consistent patterns of associations and in agreement with previous studies.

Several studies have demonstrated a link between increased early life exposure to Mn and adverse effects on mental development and cognition, including reduced IQ, learning difficulties, and increased risk of behavioral problems such as ADHD (Claus Henn et al., 2010; Farias et al., 2010; Menezes-Filho et al., 2011; Chung et al., 2015). However, other studies have not reported a link between blood Mn concentrations and behavior (Khan et al., 2011; Bhang et al., 2013) and evidence of Mn causing behavioral problems has not been conclusive (Sanders et al., 2015). We observed a consistent pattern of higher blood Mn concentrations apparently inducing an adverse effect on neurodevelopment (e.g., lower scoring for IQ, increased sway, and higher scoring for behavioral problems), which was particularly evident for behavioral outcomes. Despite increased environmental exposure, current blood Mn concentrations among the children assessed in this study were in the normal range, raising the question of whether these levels are in fact possibly neurotoxic, or are a surrogate for altered neuronal Mn homeostasis, which causes neurotoxicity *in utero* and during early postnatal development, when blood Mn levels are higher than in later childhood (Zota et al., 2009).

SLC39A8 is a cell membrane located metal transporter, responsible for Mn uptake and reabsorption in the kidney (He et al., 2006), and recovery of Mn from the bile (Lin et al., 2017). Its expression pattern also suggests that it may be involved in the uptake of Mn through the lungs and nasal respiratory epithelium (Genter et al., 2009). Mutations in the conserved sequences of *SLC39A8* cause substantial reduction in Mn concentrations and severe phenotypes with intellectual disability and developmental delay (Boycott et al., 2015), cranial asymmetry, and dwarfism (Park et al., 2015). *SLC39A8* rs13107325 has a substitution of alanine to threonine, which may reduce protein function by affecting protein formation (Zhang et al., 2016), as well as reduced *SLC39A8* expression in liver (Speliotes et al., 2010). The *SLC39A8* rs13107325 SNP has been associated with blood Mn (Ng et al., 2015) and neurological/mental conditions such as Parkinson's disease, stress, and schizophrenia (Carrera et al., 2012; Bruenig et al., 2014; Pickrell et al., 2016). Our findings of lower blood Mn concentrations in association with the rs13107325 minor allele are consistent with previous studies (Ng et al., 2015) and fit a model of the variant causing reduced protein function and consequently reduced Mn uptake (Figure 5).

We also observed associations of rs13107325 with behavioral problems and the minor allele (lower Mn) was associated with reduced scoring in the tests. In contrast, rs13107325 appeared to have less influence on IQ, despite the fact that the three rs13107325 variant allele homozygotes showed the lowest Mn in the cohort. Atypically low blood Mn has previously been associated with intellectual disability and reduced neurodevelopment (Claus Henn et al., 2010; Boycott et al., 2015; Chung et al., 2015) and a new study has also linked low Mn during development to autism (Arora et al., 2017). We cannot however rule out the possibility that our findings for rs13107325 are influenced by residual confounding as this variant has been shown to be strongly associated with childhood BMI and extreme early-onset obesity, as well as with a number of other traits (Wheeler et al., 2013; Felix et al., 2016). We observed that the Mn-increasing allele of rs13107325 was associated with lower BMI and adjustment for BMI slightly strengthened the associations of this genotype with ADHD-index and DSM-IV total. Furthermore, although several *in vitro* studies suggest that Mn is the principal substrate of SLC39A8 (He et al., 2006; Eun-Kyung, 2018; Haller et al., 2018) and mutations/variants in *SLC39A8* have predominantly been linked to variations in Mn concentrations *in vivo* (Ng et al., 2015; Park et al., 2015; Haller et al., 2018), SLC39A8 may also be involved in the transport of Zn, Cd, and Fe (He et al., 2006; Wang et al., 2012). We did not observe an association between rs13107325 and Fe in blood, however, since Zn and Cd were not measured for the children, we cannot exclude the possibility that at least some of the associations between rs13107325 and neurological outcomes could be mediated by changes in physiological concentrations of these metals.

*SLC30A10* is highly expressed in the liver and since deleterious mutations cause Mn accumulation in the bile (Tuschl et al., 2012), SLC30A10 may be involved in Mn excretion via the bile. However, *SLC30A10* is also highly expressed in the CNS and may thus exert a second level of regulation by controlling Mn homeostasis in the brain. Due to the lack of coding SNPs in *SLC30A10* with sufficiently high MAF, we focused on non-coding SNPs that potentially influence gene expression rather than protein function. We have previously shown that the rs2275707 minor allele, which is associated with higher blood Mn concentrations, is also associated with lower *SLC30A10* gene-expression in blood (Wahlberg et al., 2016). The influence of rs12064812 on *SLC30A10* gene-expression is demonstrated by publicly available expression data from the GTEx portal (https://gtexportal.org/home/, accessed October 2018) which shows that the SNP influences *SLC30A10* gene-expression in different directions dependent on tissue type. This indicate that *SLC30A10* gene-expression data in blood in relations to SNPs may not reflect the influence of SNPs on gene-expression on tissues with more relevance for Mn regulation and toxicity such as liver and brain.

Here we show that rs1776029 is strongly linked to rs2275707 and situated in a genomic region several kilo-bases downstream of the *SLC30A10* gene, which displays distinct patterns of regulatory activity making it a strong candidate to be the causative SNP. Similar patterns have previously been associated with enhancer elements involved in long-range genomic interactions with gene promoters (Wahlberg et al., 2009). A proposed model is that rs1776029 may interfere with a long-range regulatory element, which causes altered *SLC30A10* expression, and thus impaired Mn efflux capacity in the liver and brain, with a negative influence on neurodevelopment as a consequence (the opposite pattern for rs12064812; Figure 5).

The limitations of this study should be noted. Despite the use of IV analysis to infer causality, the study is cross-sectional. The analysis relies on blood Mn as a biomarker of internal dose, despite its short half-life in circulation (Zheng et al., 2000). Another limitation, as mentioned previously, is the lack of blood Cd and Zn concentration measurements which would be relevant to analyze in relation to *SLC39A8* rs13107325.

In conclusion, we show that common SNPs in *SLC39A8* and *SLC30A10* are probably significant influencing factors on blood Mn concentrations and are associated with differences in neurological performance in children. Further, by using a genotype score based on these SNPs in IV analysis, we provide support for the hypothesis that Mn concentrations influence neurodevelopment. SNPs in Mn transporters are therefore important to consider further in the context of children's neurodevelopment and in studies of Mn homeostasis and toxicity.

## Author Contributions

KW conducted research, analyzed data, and wrote the paper. SG and SL analyzed data. DaP, CF, GC, and SZ conducted research. DoP, RW, DS, and RL designed the project. KB designed the project, participated in the writing of the paper, and had primary responsibility for final content.

### Conflict of Interest Statement

The authors declare that the research was conducted in the absence of any commercial or financial relationships that could be construed as a potential conflict of interest.
