# Supplementary material for: Polymorphisms in Manganese Transporters SLC30A10 and SLC39A8 Are Associated With Children's Neurodevelopment by Influencing Manganese Homeostasis
Source: Front Genet. 2018 Dec 20;9:664. doi: 10.3389/fgene.2018.00664 (PMC6307466; doi:10.3389/fgene.2018.00664)
Supplement: Supplementary file 1 [file Data_Sheet_1.pdf]

Supplementary Material for article in Frontiers in Genetics entitled:

Polymorphisms in manganese transporters SLC30A10 and SLC39A8 are associated with children's neurodevelopment by influencing manganese homeostasis

Karin Wahlberg, Stefano Guazzetti, Daniela Pineda, Susanna C Larsson, Chiara Fedrighi, Giuseppa Cagna, Silvia Zoni, Donatella Placidi, Robert O Wright, Donald R Smith, Roberto G Lucchini, Karin Broberg

(corr author; karin.broberg@ki.se, Tel:+46 (0)73 782 37 50, Division of Occupational and Environmental Medicine, Lund University, Klinikgatan 21, 221 85 Lund, Sweden and Institute of Environmental Medicine, Karolinska Institutet, Nobels väg 13, 171 65 Solna, Sweden)

Supplementary Materials and Methods

Measurements of Mn in soil

Mn in surface soil was measured *in situ* using a portable X-ray Fluorescence (XRF) instrument (Thermo Scientific Niton, model XL3t) in the yards of the children's homes as previously described (1). The average of 2–4 randomly spaced readings was calculated. For individuals who did not have a soil-containing environment in the surroundings of their house (37% of total individuals), soil Mn levels were estimated by means of stratified (by area) universal kriging (Valcamonica and Bagnolo Mella) and ordinary kriging (Lake Garda).

Biological sampling and measurements of Mn, lead, and ferritin

Sampling and preparation of blood for analyses of Mn concentrations were conducted as previously described (1). Briefly, blood was collected in Li-Heparin Sarstedt Monovette Vacutainers. Following collection, samples were maintained frozen (-20 °C) until analysis.

Manganese and lead (Pb) levels in blood were measured by magnetic sector inductively coupled plasma mass spectrometry in the Trace Metal cleanroom facility at the University of California, Santa Cruz (Thermo Element XR ICP-MS) or by Zeeman graphite furnace atomic absorption spectrometry in the Industrial Hygiene laboratory at the University of Brescia, Italy (Varian SpectrAA), as described elsewhere (2-4).

Ferritin was measured in plasma by chemiluminescent microparticle immunoassays (CMIA) using the Architect SR 2000 Immunoassay Analyzer (Abbott Diagnostics, Illinois, USA).

Assessment of cognitive behavioral functions

The children were assessed in the morning in their local school by trained neuro-psychologists.

Intelligence- Children's IQ was assessed using the Wechsler Intelligence Scale for Children (WISC), third edition. The test includes a panel of subtests (picture completion, information, coding, similarities, picture arrangement, arithmetic, block design, vocabulary object assembly comprehension, and digit span) and gives an overall estimation of the child's general IQ but can also be subdivided into verbal IQ and performance IQ. Verbal IQ score is based on Information, Similarities, Arithmetic, Vocabulary, and Comprehension. Performance (non-verbal) IQ score is based on Picture Completion, Coding, Picture Arrangement, Block Design, and Object Assembly. Full Scale IQ score is based on the ten tests included in the Verbal and Performance (nonverbal) IQ. Raw scores of each scale are corrected for age in weighted scores from 1 (worse score) to 19 (best score).

Motor function- Children's motor function was assessed by a panel of tests covering different aspects of motor function, as previously described in detail (1, 5). Motor function was measured using 5 subtests of the Luria-Nebraska Motor Battery: dominant hand clench, non-dominant hand clench, alternative hand clench and finger-thumb touching with dominant hand and non-dominant hand. The sum of scores from each subtest is used to create a total Luria motor score (Luria sum). Psychomotor speed was assessed using a computerized version of finger tapping from the Swedish Performance Evaluation System (SPES) (6) in which the participant tapped a button within a 5-minute period, alternatively with the dominant and non-dominant hand. Reaction time was evaluated by the SPES version of Visual Simple Reaction Time Test, in which the subject was asked to press a button in response to a visual stimulus on a computer screen.

Sway/tremor- Children's tremor was assessed with Tremor 7.0 of Danish Products developments-DPD (7), in which each subject held a stylus for 10 s, during which hand vibrations were measured in a time axis plot. Body sway was measured by using a balance plate on which the sway can be monitored as the change in position from a force center in an X-Y

coordinate system. Measurements evaluated included sway area (the area of the smallest polygon in mm^2), sway intensity (the root mean square of accelerations in Hz), sway velocity (the average travel speed of the force center in the horizontal force plate plane in mm/s), and mean sway (the simple mean of the distance from the geometrical mean force center position to all recorded force center positions during the test in mm). The test was repeated with the subject's eyes open and closed.

Hand dexterity and perceptual speed was assessed using the Pursuit Aiming test (8), which included the task of quickly placing a dot with a pen within a small circle.

Behavior- Children's behavior was assessed using the Conners' Adolescent Self-Report Scale (CASS), which includes subscales created through factor analyses to assess a broad range of significant behavior problems. The test includes 87 items that yield scores for 8 subscales concerning adolescent behavior and is recommended for adolescents from 12 to 17 years old. Adolescents rate their feelings and behavior over the past month on a 4-point Likert scale. The following 10 subscales are assessed: family problems, emotional problems, conduct problems, cognitive problems/inattention, anger control problems, hyperactivity, attention deficit hyperactivity disorder (ADHD) index, DSM-IV (inattention), DSM-IV (hyperactivity/impulsivity), and DSM-IV Total (total score of the 2 subscales assessing inattention and hyperactivity/impulsivity) (9). Of the DSM-IV scales, only DSM-IV total was included in this study for assessment of ADHD-related behavior (10).

In addition to the self-reported scales, we also included revised short versions of the Conners' Parent's Rating Scales (CPRS-R) and Teachers Rating Scales (CTRS-R). CPRS-R and CTRS-R were only assessed in the second round of recruitment and scores were therefore available for approximately half of the cohort. The 27-question CPRS-R and CTRS-R included four subscales (oppositional problems, cognitive problems/inattention, hyperactivity, and ADHD-

index) and were designed to obtain the parents' and teacher's reports on behavioral problems in children of 3–17 years old, with a good test-retest reliability and internal consistency (11, 12).

Raw scores were corrected for age and sex and converted into T-scores, which are standardized scores with a mean of 50 and a standard deviation of 10. As a rule, T-scores above 56 indicate a borderline problematic picture and scores over 60 are cause for concern. The validated Italian versions of Conner's Rating Scale-Revised (CPRS-R, CTRS-R, CASS) were used for the assessment (13).

References

1. Lucchini RG, Guazzetti S, Zoni S, Donna F, Peter S, Zacco A, Salmistraro M, Bontempi E, Zimmerman NJ, Smith DR. Tremor, olfactory and motor changes in Italian adolescents exposed to historical ferro-manganese emission. Neurotoxicology 2012;33:687-96.
2. Apostoli P, Lucchini R, Alessio L. Are current biomarkers suitable for the assessment of manganese exposure in individual workers? Am J Ind Med 2000;37:283-90.
3. Smith D, Gwiazda R, Bowler R, Roels H, Park R, Taicher C, Lucchini R. Biomarkers of Mn exposure in humans. Am J of Ind Med 2007;50:801-11.
4. Lucas EL, Bertrand P, Guazzetti S, Donna F, Peli M, Jursa TP, Lucchini R, Smith DR. Impact of ferromanganese alloy plants on household dust manganese levels: implications for childhood exposure. Environ Res 2015;138:279-90.
5. Chiu YM, Claus Henn B, Hsu HL, Pendo MP, Coull BA, Austin C, Cagna G, Fedrighi C, Placidi D, Smith DR et al. Sex differences in sensitivity to prenatal and early childhood manganese exposure on neuromotor function in adolescents. Environ Res 2017;159:458-65.

6. Iregren A, Gamberale F, Kjellberg A. SPES: a psychological test system to diagnose environmental hazards. Swedish Performance Evaluation System. Neurotoxicol Teratol 1996; 18:485-91.
7. Despres C, Lamoureux D, Beuter A. Standardization of a neuromotor test battery: the CATSYS system. Neurotoxicology 2000;21:725-35.
8. Fleishman, E. A. Dimensional analysis of psychomotor abilities. J Exp Psychol 1954;48:437-54.
9. Lucchini RG, Zoni S, Guazzetti S, Bontempi E, Micheletti S, Broberg K, Parrinello G, Smith DR. Inverse association of intellectual function with very low blood lead but not with manganese exposure in Italian adolescents. Environ Res 2012;118:65-71.
10. Wolraich M, Brown L, Brown RT, DuPaul G, Earls M, Feldman HM, Ganiats TG, Kaplanek B, Meyer B, Perrin J et al. ADHD: clinical practice guideline for the diagnosis, evaluation, and treatment of attention-deficit/hyperactivity disorder in children and adolescents. Pediatrics 2011;128:1007-22.
11. Conners CK, Sitarenios G, Parker JD, Epstein JN. The revised Conners' Parent Rating Scale (CPRS-R): factor structure, reliability, and criterion validity. J Abnorm Child Psychol 1998;26:257-68.
12. Conners CK, Sitarenios G, Parker JD, Epstein JN. Revision and restandardization of the Conners Teacher Rating Scale (CTRS-R): factor structure, reliability, and criterion validity. J Abnorm Child Psychol 1998;26:279-91.
13. Conners K. Conners rating scale revised, Organizzazioni Speciali edn: Giunti O.S.; 2001.

Supplemental Table S1. Associations of genotypes with blood manganese (Mn) concentrations

		rs1776029		rs2275707		rs12064812		rs13107325		
**Out-come**	**Descriptive**	**GA vs GG**	**AA vs GG**	**CA vs AA**	**CC vs AA**	**TC vs TT**	**CC vs TT**	**CT vs CC**	**TT vs CC**	**TT/CT vs CC**
**Blood Mn**	**B[a] (CI)**	0.07 (0.05, 0.09)	0.14 (0.10, 0.18)	0.06 (0.04, 0.08)	0.10 (0.06, 0.15)	-0.03 (-0.05, -0.01)	-0.02 (-0.06, 0.02)	-0.07 (-0.09, -0.04)	-0.32 (-0.46, -0.18)	-0.07 (-0.10, -0.05)
	p	<0.001		<0.001		0.008		<0.001		<0.001

[a] Values derived from a linear regression model: log blood Mn=$\alpha+\beta_1$ genotype+ β_2 gender+ β_3 age+ β_4 ferritin+β_5 log soil Mn.

Supplementary Table S2. Overview of children's test results (weighted scores) in the Wechsler Intelligence Scale for Children.

Test	N	Median	Percentile	
			5th	95th
**Picture completion**	681	11	7	16
**Information**	681	12	7	16
**Coding**	681	11	6	16
**Similarities**	681	11	7	15
**Picture arrangement**	681	11	6	16
**Arithmetic**	681	11	5	15
**Block design**	681	12	7	16
**Vocabulary**	681	10	7	15
**Object assembly**	681	12	7	16
**Comprehension**	681	10	6	14
**Digit span**	681	11	6	15
**Verbal IQ**	681	104	81	124
**Performance IQ**	681	109	87	128
**Total IQ**	681	108	85	127

Supplementary Table S3. Overview of children's results in tests assessing motor function, reaction time, hand dexterity, tremor, and sway.

Test	N	Median	Percentile	
			5th	95th
**Luria total score**	680	63.0	43.1	89.0
**Finger tapping DH**	678	60.0	50.0	68.0
**Finger tapping NDH**	678	51.0	41.0	61.0
**Aiming**	683	125.0	82.2	172.6
**Reaction time**	679	302.0	249.0	392.0
**Tremor intensity L**	681	0.2	0.1	0.5
**Sway Area CE**	680	401.0	113.2	1213.4
**Sway Intensity CE**	680	5.1	2.9	9.0
**Sway velocity CE**	680	13.4	6.7	24.6
**Mean sway CE**	680	6.1	3.3	10.7

Abbreviations: Dominant hand, DH; non-dominant hand, NDH; right hand, R; left hand, L; open eyes, OE; closed eyes, CE

Supplementary Table S4. Overview of children's scoring (T-scores) in the Conners' Rating Scales.

Main scale	Subscale	*N*	Median	Percentile	
				5th	95th
Conners' Adolescents Self-Report Scale- Long Form	Family problems	680	42	35	61
	Emotional problems	680	44	36	64
	Conduct problems	680	44	39	56
	Cognitive problems/inattention	680	44	36	64
	Anger control problems	680	43	35	63
	Hyperactivity	680	46	35	70
	ADHD-index	680	42	36	63
	DSM-IV total	680	45	36	63
Conners' Parents Rating Scale- Short Form	Oppositional	382	47	36	73
	Cognitive problems/ inattention	382	44	41	63
	Hyperactivity	382	45	40	63
	ADHD-index	382	47	39	75
Conners' Teachers Rating Scale- Short Form	Oppositional	270	45	42	58
	Cognitive problems/ inattention	270	45	40	69
	Hyperactivity	270	45	39	59
	ADHD-index	270	44	37	65

Supplementary Table S5. Cohort characteristics of variables adjusted for in statistical models evaluating associations of genotypes with neurological parameters.

**Variable**		***n***	**%**
**SES**	Low	158	23.0
	Medium	359	52.3
	High	155	22.6
**Maternal education**	Low	281	42.0
	Medium	295	44.1
	High	93	13.9
**Parity**	1	11	14.6
	2	414	60.9
	3	131	19.3
	4	32	4.7
	5	3	0.4
	6	1	0.1
**Alcohol intake**	Yes	25	3.7
	No	657	96.3

Abbreviations: SES = Socioeconomic Status

Supplementary Table S6. Associations of genotypes and blood Mn concentrations with neurological function including IQ, motor function, and balance. Associations of blood Mn with neurological outcomes are presented for ordinary linear regression models.

**Neurological test**		**rs1776029** GA vs GG	**rs1776029** AA vs GG	**rs12064812** TC vs TT	**rs12064812** CC vs TT	**rs13107325** CT/TT vs CC
Picture completion	β^{a} (CI)	0.13 (-0.35, 0.61)	-1.18** (-2.24, -0.11)	0.31 (-0.15, 0.76)	0.16 (-0.66, 0.98)	0.16 (-0.43, 0.74)
	P^{b}	0.113		0.598		0.311
Information	β^{a} (CI)	-0.41* (-0.85, 0.04)	-0.44 (-1.43, 0.55)	0.26 (-0.16, 0.68)	0.66* (-0.10, 1.42)	0.05 (-0.49, 0.60)
	P^{b}	0.188		0.299		0.809
Coding	β^{a} (CI)	-0.19 (-0.65, 0.27)	-0.97* (-2.01, 0.07)	0.42* (-0.02, 0.86)	0.96** (0.17, 1.76)	0.36 (-0.20, 0.93)
	P^{b}	0.429		0.053		0.348
Similarities	β^{a} (CI)	0.08 (-0.32, 0.48)	-0.31 (-1.20, 0.59)	0.11 (-0.27, 0.49)	0.28 (-0.41, 0.96)	0.27 (-0.21, 0.76)
	P^{b}	0.893		0.845		0.361
Picture arrangement	β^{a} (CI)	0.16 (-0.38, 0.70)	0.02 (-1.19, 1.23)	-0.27 (-0.78, 0.24)	0.53 (-0.40, 1.46)	0.08 (-0.58, 0.74)
	P^{b}	0.738		0.291		0.806
Arithmetic	β^{a} (CI)	-0.66** (-1.20, -0.13)	-1.21** (-2.41, -0.01)	0.30 (-0.22, 0.81)	0.35 (-0.57, 1.28)	0.40 (-0.25, 1.06)
	P^{b}	0.006		0.491		0.186
Block design	β^{a} (CI)	0.15 (-0.31, 0.61)	-0.03 (-1.06, 1.00)	0.18 (-0.26, 0.61)	0.31 (-0.47, 1.10)	0.05 (-0.52, 0.61)
	P^{b}	0.829		0.745		0.909
Vocabulary	β^{a} (CI)	0.37* (-0.05, 0.79)	0.12 (-0.82, 1.05)	1.20 (-0.20, 0.60)	-0.44 (-1.16, 0.29)	0.12 (-0.39, 0.63)
	P^{b}	0.139		0.071		0.944
Object assembly	β^{a} (CI)	0.13 (-0.36, 0.62)	0.36 (-0.74, 1.45)	-0.05 (-0.52, 0.41)	-0.28 (-1.11, 0.56)	0.04 (-0.56, 0.64)
	P^{b}	0.566		0.692		0.943
Comprehension	β^{a} (CI)	-0.05 (-0.42, 0.33)	-0.30 (-1.14, 0.53)	0.03 (-0.33, 0.38)	-0.09 (-0.74, 0.55)	-0.18 (-0.63, 0.28)
	P^{b}	0.866		0.629		0.177
Digit span	β^{a} (CI)	-0.67*** (-1.16, -0.19)	-0.90 (-1.97, 0.18)	0.36 (-0.10, 0.82)	1.06***(0.23, 1.89)	-0.31 (-0.91, 0.28)
	P^{b}	0.011		0.053		0.816
Verbal IQ	β^{a} (CI)	-1.30 (-3.46, 0.86)	-3.25 (-8.09, 1.58)	1.36 (-0.70, 3.42)	1.14 (-2.58, 4.86)	0.51 (-2.14, 3.15)
	P^{b}	0.343		0.417		0.821
Perform. IQ	β^{a} (CI)	0.41 (-1.82, 2.64)	-2.45 (-7.43, 2.53)	1.12 (-0.98, 3.23)	2.52 (-1.29, 6.32)	1.14 (-1.58, 3.86)

	P^b	0.622		0.588		0.402
Total IQ	β^a (CI)	-0.26 (-2.42, 1.90)	-2.76 (-7.60, 2.07)	0.95 (-1.11, 3.01)	1.88 (-1.83, 5.60)	0.83 (-1.82, 3.47)
	P^b	0.713		0.751		0.634
Luria sum	β^a (CI)	2.09* (-0.26, 4.44)	-6.10**(-11.48, -0.99)	-1.59 (-3.84, 0.66)	-1.83 (-5.84, 2.19)	0.58 (-2.31, 3.47)
	P^b	0.004		0.335		0.998
Finger tapping (NDH)	β^a (CI)	0.47 (-0.56, 1.50)	-0.82 (-3.15, 1.51)	0.21 (-0.76, 1.17)	1.97** (0.22, 3.71)	0.63 (-0.62, 1.88)
	P^b	0.378		0.053		0.642
Aiming	β^a (CI)	3.14 (-3.29, 9.57)	0.65 (-13.73, 15.03)	2.12 (-3.98, 8.22)	6.95 (-3.99, 17.88)	4.17 (-3.68, 12.02)
	P^b	0.361		0.375		0.778
Reaction time	β^a (CI)	3.14 (-4.73, 11.00)	4.70 (-12.84, 22.24)	-3.10 (-10.52, 4.33)	-1.79 (-15.19, 11.61)	-2.49 (-12.06, 7.08)
	P^b	0.516		0.956		0.841
Tremor intensity (L)	β^a (CI)	-0.01 (-0.04, 0.03)	0.01 (-0.06, 0.09)	0.02 (-0.01, 0.05)	-0.01 (-0.06, 0.05)	-0.02 (-0.06, 0.02)
	P^b	0.261		0.206		0.789
Sway area (CE)	β^a (CI)	-0.02 (-0.08, 0.04)	0.04 (-0.09, 0.18)	-0.08***(-0.14, -0.02)	-0.09* (-0.20, 0.01)	-0.06* (-0.14, 0.02)
	P^b	0.502		0.010		0.063
Sway velocity (CE)	β^a (CI)	-0.003 (-0.04, 0.03)	0.02 (-0.06, 0.09)	-0.04** (-0.07, -0.01)	-0.03 (-0.09, 0.03)	-1.04* (-0.08, 0.01)
	P^b	0.882		0.048		0.095

Abbreviations: Non-dominant hand, NDH; left hand, L; closed eyes, CE

[a] Asterisks refer to *p*-values of associations between genotypes (*=p<0.10; **p<0.05; ***p<0.01)

[b] *p*-values of associations between genotypes and outcome refers to overall *p*-value of genotype variable.

Supplementary Table S7. Associations of genotypes and blood Mn with Conners' CASS, CPRS, and CTRS test results. Associations of blood Mn with behavioral outcomes are presented for ordinary linear regression.

Main scale	Subscale	Para meter	rs1776029 GA vs GG	rs1776029 AA vs GG	rs12064812 TC vs TT	rs12064812 CC vs TT	rs13107325 CT/TT vs CC
CASS	Family problems	β^a (CI)	0.004 (-1.01, 0.02)	0.01 (-0.02, 0.05)	-0.01 (-0.02, 0.005)	-0.01 (-0.03, 0.02)	-0.02** (-0.04, -0.004)
		P^b	0.503		0.370		0.006
	Emotional problems	β^a (CI)	0.005 (-0.01, 0.02)	0.03 (-0.01, 0.06)	-0.01 (-0.02, 0.01)	0.01 (-0.01, 0.03)	-0.01 (-0.03, 0.01)
		P^b	0.223		0.235		0.286
	Conduct problems	β^a (CI)	0.001 (-0.01, 0.01)	0.01 (-0.01, 0.03)	-0.01** (-0.02, -0.003)	-0.01 (-0.03, 0.003)	-0.01* (-0.02, 0.0001)
		P^b	0.581		0.029		0.058
	Cognitive problems/ inattention	β^a (CI)	0.01 (-0.01, 0.02)	0.02 (-0.01, 0.05)	-0.01 (-0.02, 0.01)	-0.004 (-0.03, 0.02)	-0.01 (-0.03, 0.01)
		P^b	0.211		0.675		0.267
	Anger control problems	β^a (CI)	0.002 (-0.01, 0.01)	0.03* (-0.002, 0.06)	-0.01* (-0.02, 0.001)	-0.001 (-0.02, 0.02)	-0.01 (-0.02, 0.01)
		P^b	0.141		0.135		0.165
	Hyperactivity	β^a (CI)	0.01 (-0.004, 0.03)	0.01 (-0.02, 0.05)	-0.003 (-0.02, 0.01)	-0.004 (-0.03, 0.02)	-0.02** (-0.04, 0.003)
		P^b	0.260		0.847		0.039
	ADHD-index	β^a (CI)	0.01 (-0.01, 0.02)	0.03 (-0.01, 0.06)	-0.01* (-0.02, 0.002)	-0.01 (-0.03, 0.02)	-0.02*(-0.03, -0.0002)
		P^b	0.175		0.205		0.052
	DSM-IV total	β^a (CI)	0.01 (-0.002, 0.02)	0.03** (0.0003, 0.06)	-0.01 (-0.02, 0.004)	-0.01 (-0.03, 0.02)	-0.02***(-0.04, -0.004)
		P^b	0.019		0.306		0.006
CPRS	Oppositional	β^a (CI)	0.03** (0.01, 0.05)	0.02 (-0.02, 0.07)	-0.004 (-0.02, 0.02)	-0.01 (-0.05, 0.03)	0.02 (-0.003, 0.05)
		P^b	0.060		0.794		0.141
	Cognitive problems/ inattention	β^a (CI)	0.01 (-0.01, 0.03)	0.04* (-0.004, 0.08)	-0.02* (-0.03, 0.002)	-0.005 (-0.04, 0.03)	-0.01 (-0.03, 0.02)
		P^b	0.082		0.145		0.439
	Hyperactivity	β^a (CI)	0.02* (-0.001, 0.03)	0.001 (-0.03, 0.04)	-0.01 (-0.02, 0.004)	-0.02 (-0.04, 0.01)	-0.002 (-0.02, 0.02)
		P^b	0.211		0.199		0.723

	ADHD-index	β[a] (CI)	0.02** (0.0001, 0.04)	0.03 (-0.02, 0.08)	-0.01 (-0.03, 0.01)	-0.01 (-0.05, 0.03)	0.003 (-0.02, 0.03)
		P[b]	0.138		0.611		0.963
CTRS	Oppositional	β[a] (CI)	0.01 (-0.01, 0.03)	0.02 (-0.02, 0.05)	-0.01 (-0.03, 0.004)	-0.03** (-0.06, -0.000)	-0.01 (-0.03, 0.01)
		P[b]	0.624		0.129		0.246
	Cognitive problems/ inattention	β[a] (CI)	0.01 (-0.01, 0.04)	0.01 (-0.04, 0.05)	-0.004 (-0.02, 0.01)	-0.01 (-0.05, 0.02)	-0.02 (-0.05, 0.002)
		P[b]	0.761		0.924		0.138
	Hyperactivity	β[a] (CI)	0.02** (0.002, 0.04)	0.01 (-0.03, 0.05)	-0.01 (-0.03, 0.005)	-0.03* (-0.07, 0.002)	-0.02 (-0.04, 0.001)
		P[b]	0.230		0.163		0.102
	ADHD-index	β[a] (CI)	0.02** (0.003, 0.04)	0.01 (-0.04, 0.05)	-0.01 (-0.03, 0.01)	-0.03* (-0.07, 0.01)	-0.02 (-0.04, 0.01)
		P[b]	0.294		0.389		0.259

Abbreviations: CASS, Conners' Adolescent Self-Report Scale; CPRS, Conners' Parent's Rating Scales; CTRS Conners' Teachers's Rating Scales; OLS, ordinary least squares; IV, instrumental variable; CI, confidence interval; ADHD, attention deficit hyperactivity disorder, ADHD; DSM-IV total, Diagnostic and Statistical Manual of Mental Disorders, 4th Edition total score of inattention and hyperactivity/impulsivity.

[a] Asterisks refer to *p*-values of associations between genotypes. p<0.10; **p<0.05; ***p<0.01

[b] *p*-values of associations between genotypes and outcome refers to overall *p*-value of genotype variable.

Supplementary Table S8. Instrument variable analysis of genotype score and blood Mn concentrations with neurological functions including IQ, motor function, and balance.

Neurological test	Statistic	OLS	IV
		Blood Mn	Blood Mn
Picture completion	β (CI)	-0.13 (-1.86, 1.60)	-3.93 (-9.21, 1.35)
	P[a]	p=0.88	p=0.145
Information	β (CI)	1.77** (0.16, 3.38)	-3.10** (-8.08, 1.87)
	P[a]	p=0.031	p=0.22
Coding	β (CI)	1.16 (-0.52, 2.85)	-6.35*** (-11.73, -0.97)
	P[a]	p=0.177	p=0.021
Similarities	β (CI)	0.64 (-0.80, 2.08)	-1.40 (-5.75, 2.96)
	P[a]	p=0.387	p=0.529
Picture arrangement	β (CI)	-0.10 (-2.04, 1.84)	1.22 (-4.61, 7.06)
	P[a]	p=0.92	p=0.681
Arithmetic	β (CI)	0.17 (-1.80, 2.14)	-7.92*** (-14.16, -1.68)
	P[a]	p=0.868	p=0.013
Block design	β (CI)	1.29 (-0.39, 2.96)	0.31 (-4.72, 5.34)
	P[a]	p=0.132	p=0.904
Vocabulary	β (CI)	1.07 (-0.44, 2.57)	1.74 (-2.79, 6.27)
	P[a]	p=0.167	p=0.452
Object assembly	β (CI)	1.16 (-0.63, 2.94)	2.95 (-2.42, 8.32)
	P[a]	p=0.204	p=0.282
Comprehension	β (CI)	0.61 (-0.74, 1.97)	0.45 (-3.62, 4.51)
	P[a]	p=0.375	p=0.83
Digit span	β (CI)	0.17 (-1.60, 1.94)	-6.57*** (-12.14 -1.01)
	P[a]	p=0.852	p=0.021
Verbal IQ	β (CI)	5.85 (1.99, 13.69)	-15.53* (-39.65, 8.58)

	P[a]	p=0.144	p=0.207
Perform. IQ	β (CI)	4.90 (-3.15, 12.94)	-10.76 (-35.21, 13.69)
	P[a]	p=0.233	p=0.389
Total IQ	β (CI)	6.44 (-1.37, 14.25)	-9.70 (-33.49, 14.08)
	P[a]	p=0.107	p=0.424
Luria sum	β (CI)	2.28 (-6.31, 10.87)	7.41 (-18.30, 33.12)
	P[a]	p=0.604	p=0.573
Finger tapping (NDH)	β (CI)	0.46 (-3.27, 4.19)	-5.11 (-16.28, 6.06)
	P[a]	p=0.808	p=0.37
Aiming	β (CI)	17.82 (-5.52, 41.16)	1.19 (-68.80, 71.17)
	P[a]	p=0.135	p=0.974
Reaction time	β (CI)	11.24 (-17.19, 39.67)	66.40 (-19.44, 152.24)
	P[a]	p=0.439	p=0.13
Tremor intensity (L)	β (CI)	0.072 (-0.047, 0.19)	0.002 (-0.35, 0.36)
	P[a]	p=0.233	p=0.991
Sway area (CE)	β (CI)	0.020 (-0.21, 0.25)	0.75** (0.050, 1.46)
	P[a]	p=0.866	p=0.036
Sway velocity (CE)	β (CI)	-0.071 (-0.20, 0.052)	0.33** (-0.057, 0.71)
	P[a]	p=0.258	p=0.096

Abbreviations: Ordinary least squares, OLS, instrumental variable, IV; non-dominant hand, NDH; left hand, L; closed eyes, CE

[a] *p*-values of associations between genotypes and outcome refers to overall *p*-value of genotype variable. Asterisks (**p*<0.10; ***p*<0.05; ****p*<0.01) after *p*-values for IV refer to *p*-value of Wu-Hausman test where a low *p*-value indicates that the IV model is more consistent than OLS, and thus, the more suitable model of the two.

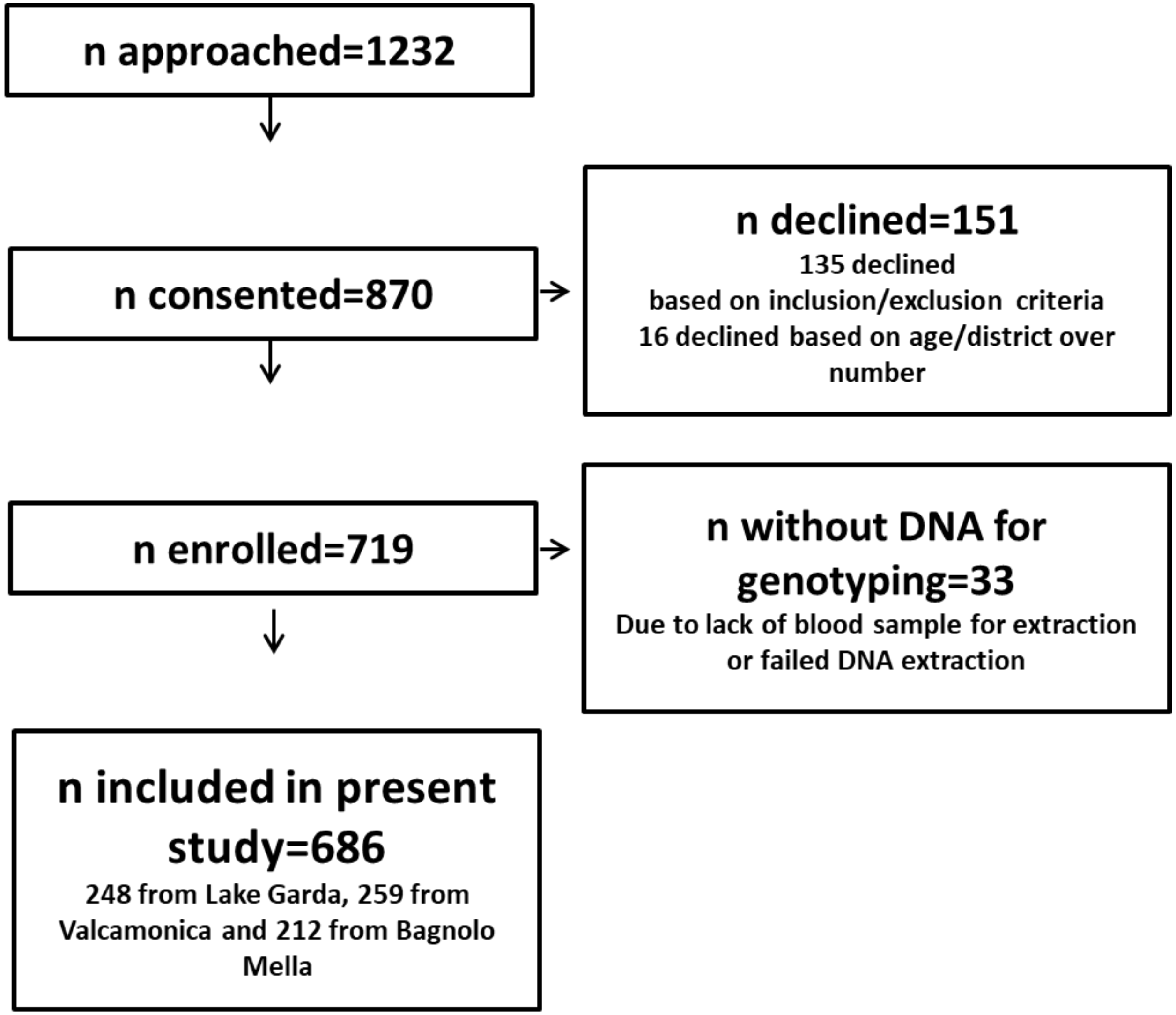

Supplementary Figure S1. Participant flowchart of study.

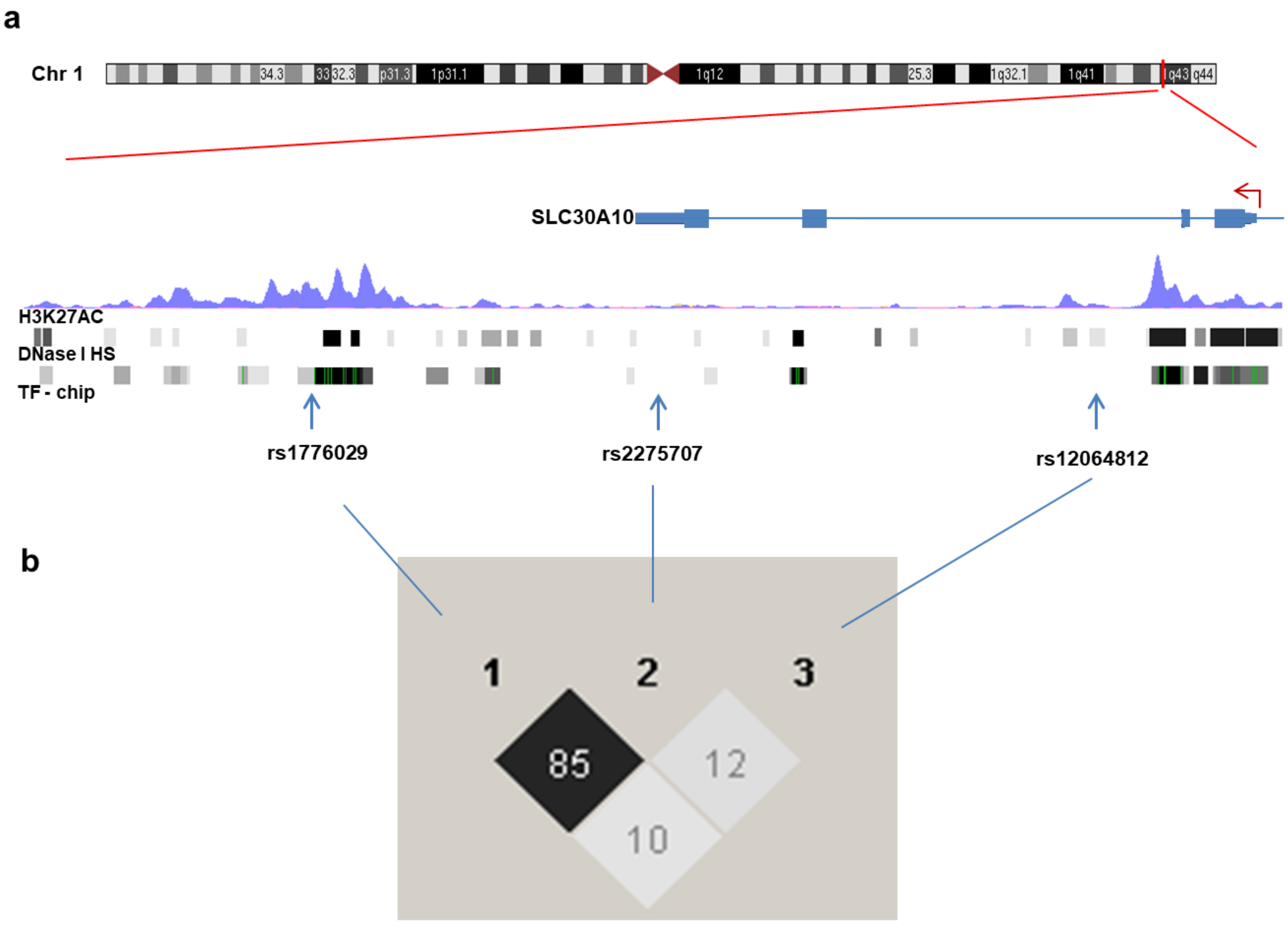

Supplementary Figure S2. A) Genetic location of SNPs in relation to the *SLC30A10* gene and signatures of gene-regulatory elements available from the UCSC Genome Browser (https://genome.ucsc.edu/) including Histone H3 acetylation at Lys27 (H3K27Ac), DNase I hypersensitive sites (DNase I) and transcription factor (TF) binding sites. B) Linkage analyses of the 3 *SLC30A10* SNPs. Linkage is presented as increased shading. Numbers represent R^2 values of correlations between SNPs.
